# No relationship between circulating levels of sex steroids and mammographic breast density: the Prospect-EPIC cohort

**DOI:** 10.1186/bcr1758

**Published:** 2007-08-13

**Authors:** Martijn Verheus, Petra HM Peeters, Paulus AH van Noord, Yvonne T van der Schouw, Diederick E Grobbee, Carla H van Gils

**Affiliations:** 1 Julius Center for Health Sciences and Primary Care, Room Str 6.131, PO Box 85500, University Medical Center Utrecht, Utrecht 3508 GA, The Netherlands

## Abstract

**Background:**

High breast density is associated with increased breast cancer risk. Epidemiologic studies have shown an increase in breast cancer risk in postmenopausal women with high levels of sex steroids. Hence, sex steroids may increase postmenopausal breast cancer risk via an increase of breast density. The objective of the present study was to study the relation between circulating oestrogens and androgens as well as sex hormone binding globulin (SHBG) in relation to breast density.

**Methods:**

We conducted a cross-sectional study among 775 postmenopausal women, using baseline data of a random sample of the Prospect-EPIC study. Prospect-EPIC is one of two Dutch cohorts participating in the European Prospective Investigation into Cancer and Nutrition, and women were recruited via a breast cancer screening programme. At enrolment a nonfasting blood sample was taken and a mammogram was made. Oestrone, oestradiol, dehydroepiandrosterone sulfate, androstenedione, testosterone and SHBG levels were measured, using double-antibody radioimmunoassays. Concentrations of free oestradiol and free testosterone were calculated from the measured oestradiol, testosterone and SHBG levels Mammographic dense and nondense areas were measured using a semiquantitative computerized method and the percentage breast density was calculated. Mean breast measures for quintiles of hormone or SHBG levels were estimated using linear regression analyses.

**Results:**

Both oestrogens and testosterone were inversely related with percent breast density, but these relationships disappeared after adjustment for BMI. None of the sex steroids or SHBG was associated with the absolute measure of breast density, the dense area.

**Conclusion:**

The results of our study do not support the hypothesis that sex steroids increase postmenopausal breast cancer risk via an increase in breast density.

## Introduction

Dense breast tissues (glandular and stromal tissues) appear light on a mammogram, while nondense tissue (fat tissue) appears black [1]. Women with a high percentage of breast density have been shown to have strongly increased risk for breast cancer development [2,3].

In epidemiologic studies, nulliparity, late age at first child birth and late age of menopause have all been related to increased breast density, indicating that sex steroid levels may influence breast density; it has therefore been hypothesized that breast density may reflect lifetime exposure to sex steroids. Moreover, postmenopausal hormone therapy (HT), and in particular combination therapy of oestrogens and progesterone, increases breast density in most women [4-7], whereas Tamoxifen, an 'anti-oestrogen', decreases breast density [8,9]. Reproductive factors as well as HT use have been shown to have the same positive associations with breast cancer risk as described with breast density [10,11].

Higher circulating levels of oestrogens and androgens have been clearly established to lead to higher breast cancer risk in postmenopausal women [12,13]. In the light of the above-described relationships between reproductive factors and breast density, and also exogenous hormones and breast density, one would also expect that circulating levels of endogenous sex steroids increase breast cancer risk by altering breast density. Greendale and colleagues indeed showed that women with high oestradiol levels had significantly increased percentage breast density [14] Boyd and colleagues, however, found a statistically significant inverse relationship between oestradiol levels and percentage density [15]. Three other studies did not find oestrogens and breast density to be associated [16-18]. Androgens were not related to breast density in any of these studies, but most studies reported higher breast density with higher sex hormone binding globulin (SHBG) levels, which appears to contradict the inverse relation between SHBG levels and postmenopausal breast cancer risk shown in many studies [12,13].

Most of the studies on sex steroids and breast density were small, however (range, 88–1413 subjects), and contained a relatively large percentage of former HT users (range, 30–50%). It is not known what time period is needed for a complete washout of HT effects on breast tissue, and hence results may be influenced. Furthermore, four of these studies [14,16-18], but not that of Boyd and colleagues [15], present results on percentage breast density only. The dense area may, however, be a better marker of breast cancer risk than the percentage breast density, as it is the absolute amount of glandular and stromal (dense) tissue that is regarded as the target tissue for breast cancer.

In the present large, cross-sectional study we set out to investigate the relation between circulating levels of oestrogens, androgens and SHBG, on the one hand, and both measures of breast density (dense area and percentage density), on the other, in a sample of Dutch women, with very few (14%) former HT users.

## Methods

### Prospect cohort

This study included women participating in Prospect-EPIC, one of two Dutch cohorts participating in the European Prospective Investigation into Cancer and Nutrition (EPIC). EPIC is a multicentre cohort study with 10 participating European countries. The rationale and design of both the EPIC and Prospect-EPIC have been described in detail elsewhere [19-21]. The Prospect-EPIC cohort consists of 17,357 women, aged 49–69 years at enrolment. Women were recruited between 1993 and 1997 through a regional programme for breast cancer screening and reside in Utrecht or its vicinity. The regional programme is part of the national screening programme that covers the entire Dutch population. As part of the population-based screening programme, mammographic examinations are carried out every 2 years, starting at age 50 until age 75, and are stored in archives.

An invitation to join the Prospect-EPIC project was mailed to women that were invited for their routine mammography. Those who agreed to participate subsequently received two questionnaires by mail. A general questionnaire was used to gather information on demographic, reproductive and lifestyle factors and on past and current morbidity. To determine the regular dietary intake, a validated extensive self-administered food frequency questionnaire was used containing 178 food items [22,23]. On the scheduled breast screening date, subsequent to mammographic examination, anthropometric measures were taken and a blood sample was drawn and stored at -196°C under liquid nitrogen.

All participants signed an informed consent and the study was approved by The Institutional Review Board of the University Medical Center Utrecht.

### Study population

A 10% random sample (*n *= 1,736) was taken from the total Prospect cohort. Mammograms of 1,595 women could be retrieved from the archives and breast density was successfully measured for 1,516 women. Of these, premenopausal or perimenopausal women or those having an unknown menopausal status (*n *= 377) were excluded. Women with unknown menopausal status due to hysterectomy were also excluded (*n *= 257). Women with a hysterectomy after natural menopause (*n *= 4) and women with ovariectomy on both ovaries were kept in the study (*n *= 60). None of the 882 remaining women were current HT or oral contraceptive users. For 775 of these women, sex steroid and SHBG levels were available that were used in a previous study [24]. For 98.3% of the women in the present study, the measurement of anthropometric factors, the mammographic examination and the blood draw were performed on the same day.

### Blood collection and measurements of sex steroids and SHBG in plasma

A 30 ml nonfasting blood sample was donated by each participant, using three safety monovettes – one dry monovette for serum and two citrated monovettes for plasma. Within 24 hours, samples of 4 ml serum, 9 ml citrate plasma and 2 ml white blood cells were fractionated into 0.5 ml aliquots and were stored in heat-sealed plastic straws under liquid nitrogen at -196°C.

Plasma levels of oestrone, oestradiol, androstenedione, dehydroepiandrosterone sulfate (DHEAS), testosterone and SHBG were measured using commercially available double-antibody radioimmunoassay kits (Diagnostics System Laboratories Inc., Webster, TX, USA). The following kits were used: oestrone, DSL-8700; oestradiol, DSL-39100; androstenedione, DSL-4200; DHEAS, DSL-2700; testosterone, DSL-4100; and SHBG, DSL-6300. The intra-assay coefficients of variation were 5.6%, 3.9%, 4.3%, 5.2%, 7.7% and 3.0%, respectively. The inter-assay coefficients of variation were 11.1%, 4.1%, 6.3%, 5.3%, 8.1% and 4.0%, respectively [24].

Concentrations of free oestradiol and free testosterone were calculated from total plasma concentrations of oestradiol, testosterone and SHBG, using theoretical calculations described by Vermeulen and colleagues [25].

### Mammographic density analysis

The mediolateral oblique mammogram, which is the routine view for breast cancer screening in The Netherlands, was used to assess mammographic density. The proportions of mammographic density on craniocaudal views and mediolateral oblique views and on left and right views have been observed to be very strongly correlated, and representative information on mammographic density is provided in a single view [26]. For each study subject, mammographic density was assessed on the left view.

After digitizing the films using a laser film scanner (Lumiscan 50; Lumisys, Eastman Kodak Co., Rochester, NY, USA), the mammographic density was quantified using a computer-assisted method based on grey levels of pixels in the digitized mammogram. This computer-assisted method to determine mammographic density has proved very reliable, and the method is described elsewhere in detail [1]. Briefly, for each image, the reader first sets a threshold to determine the outside edge of the breast, to separate the image of the breast from the dark background surrounding it. A second threshold is set to determine the area of dense tissue within the breast, which is the lightest tissue visible on the mammogram. Before setting the thresholds, a masking tool is used to block the pectoralis muscle, to prevent including the pectoralis muscle in the breast and dense areas. The program then determines the amount of pixels within the total breast area and within the dense area. A conversion factor of 0.000256 was used to calculate the surface of the total breast area and the dense area from the number of pixels.

To compute the percentage breast density the dense area of a breast is divided by the total breast area and multiplied by 100. The percentage breast density is used in most publications on breast density. A small-sized breast and a larger-sized breast, however, could have the same percentage breast density, while the absolute amount of glandular and stromal tissue, which is regarded as the target tissue for breast cancer [27,28], is higher in the larger breast [29]. Hence, we shall present results for both relative and absolute measures of breast density.

All mammograms were assessed by one observer in sets composed of 35 randomly ordered films. To assess the reliability of the reader, a library set was created, which consisted of 35 randomly chosen films from our study subjects. This library set was read before the first set, after the last set and at four time points between sets, which were blinded for the reader. The images in the library set were randomly ordered every time they were read to prevent the observer from recognizing this set. In the present study, average intraclass correlation coefficients of 1.00 (range, 0.99–1.00), of 0.93 (range, 0.91–0.97) and of 0.91 (range, 0.87–0.94) were reached between repeated readings for the total breast area, the dense area and the percentage breast density, respectively.

### Statistical analyses

Subjects with plasma sex steroid or SHBG values under the detection limit – which was 1.2 pg/ml, 1 pg/ml, 25 ng/ml, 0.02 ng/ml, 0.05 ng/ml and 5 nmol/l for oestrone, oestradiol, DHEAS, androstenedione, testosterone and SHBG, respectively – were given the value of the detection limit. Six women had an undetectable oestrone level, five women were undetectable for DHEAS, eight women were undetectable for androstenedione, eight women were undetectable for testosterone and 60 women had an undetectable SHBG level. All women had a detectable value for oestradiol. Plasma sex steroid and SHBG levels were log-transformed to normalize the distributions. These transformed values were then used to compute geometric mean levels.

All three measures of the breast (percentage breast density, dense area and nondense area) were square-root-transformed to normalize their distributions. These transformed values were used in linear regression analyses. For ease of interpretation, the presented means and 95% confidence intervals have been transformed back to the original scale. Means and 95% confidence intervals of breast measures by quintile level of plasma sex steroids or SHBG were estimated with linear regression models using the 'GLM' procedure. To test for linear trends over the quintiles, median values within quintiles were calculated and evaluated as a continuous variable using linear regression analysis. Potential confounding of various factors with known association with sex steroid levels or with breast measures, or with both, was assessed by adding those variables to the crude models.

The following characteristics were evaluated for confounding using continuous variables: age, body mass index (BMI), waist circumference, waist–hip ratio (WHR), age at menarche, age at menopause, time since menopause and alcohol consumption. Dichotomous variables were used for ever breast feeding and for family history of breast cancer (mother and/or sister). A variable for ever use of postmenopausal HT combined with time since last use of HT was used with one category for never users and with two categories for ever users – the first of which contained women with a time period since last HT use below the median (5 years), and the second contained women with a time period since last use of the median value or greater. Parity and age at first childbirth were evaluated using a combined variable with one category for nulliparous women and three categories of parous women combined with tertiles of age at birth of the first child (tertile cutoff points, 23 and 26 years). Smoking was evaluated using a variable with three categories, for current smokers, past smokers and never smokers. Variables that changed the crude associations by 5% or more were added to the adjusted models. In a previous study by Aiello and colleagues, different effects were found between HT never users and ever users [16]. We therefore stratified the analyses of all three breast measures by HT ever/never use, for comparison.

All *P *values are two-sided, and results were considered significant when below 0.05. All analyses were conducted using the Statistical Analysis System software package (release 9.1; SAS Institute, Cary, NC, USA).

## Results

Table 1 presents the basic characteristics of the study population. The mean age at study recruitment was 60.2 years and the mean BMI was 26.0 kg/m^2^. The age at first childbirth among parous women was 25.4 years on average, and 12.8% (*n *= 99) of the participants was nulliparous. Women were aged, on average, 48.8 years at menopause, and the average time since menopause was 11.2 years. Only 14.4% (*n *= 112) of the participants had previously used HT; those previous HT users quit using these compounds on average 7.1 years prior to recruitment (range, 0–33 years). Median values for the percentage density, dense area and nondense area were 21.7% (interquartile range, 14.9–30.8%), 25.3 cm^2 ^(interquartile range, 18.4–35.5 cm^2^) and 95.1 cm^2 ^(interquartile range, 70.1–121.9 cm^2^), respectively.

**Table 1:** Basic characteristics of the study population (*n *= 775)

Age (years)	60.2 (5.4)
Height (cm)	163.7 (6.1)
Weight (kg)	69.7 (11.7)
Body mass index (kg/m^2^)	26.0 (4.1)
Waist–hip ratio	0.79 (0.06)
Family history of breast cancer (mother and/or sister with history of breast cancer)	106 (13.7)^a^
Reproductive factors	
Age at menarche (years)	13.5 (1.7)
Nulliparous	99 (12.8)^a^
Age at first child birth(years) (among parous women only)	25.4 (4.0)
Number of children (average) (among parous women only)	2.6 (0.9)
Ever breast feeding(among parous women only)	558 (82.5)^a^
Age at menopause (years)	48.8 (4.9)
Time since menopause (years)	11.2 (6.8)
Lifestyle factors	
Ever used oral contraceptives	416 (53.7)^a^
Ever used hormone therapy	112 (14.4)^a^
Time since stopped hormone therapy use (years) (among former hormone therapy users only)	7.1 (6.4)
Current smoking	171 (22.1)^a^
Ever smoked	421 (54.3)^a^
Alcohol intake (g/day)	8.1 (11.2)
Sex steroids and sex hormone binding globulin	
Oestrone (pg/ml)	15.26 (4.38–53.16)^b^
Oestradiol (pg/ml)	8.22 (2.92–23.17)^b^
Free oestradiol (pg/ml)	0.24 (0.09–0.73)^b^
Dehydroepiandrosterone sulfate (ng/ml)	446.7 (102.3–1,949.8)^b^
Androstenedione (ng/ml)	0.46 (0.10–2.11)^b^
Testosterone (ng/ml)	0.25 (0.09–0.72)^b^
Free testosterone (pg/ml)	5.30 (1.46–19.23)^b^
Sex hormone binding globulin (nmol/l)	19.63 (5.21–73.96)^b^
Breast measures	
Percentage breast density	21.7 (14.9–30.8)^c^
Dense area	25.3 (18.4–35.5)^c^
Nondense area	95.1 (70.1–121.9)^c^

Data presented as the mean (standard deviation), unless stated otherwise: ^a^*n *(%), ^b^geometric mean (± 2 standard deviations), ^c^median (interquartile range).

The results of regression analyses for the relation between sex steroids and the percentage breast density, the dense area and the nondense area are presented in Tables 2, 3 and 4, respectively. Both crude associations and associations adjusted for variables that changed associations are presented. These variables only included BMI in the models to calculate the mean percentage breast density and the mean nondense area. Adding a second marker for total body fat (WHR) to these models only had an additional effect on the models with SHBG. The WHR was therefore added to these models only (Tables 2 and 4 for the percentage density and the nondense area, respectively). None of the potential confounders that were tested influenced associations between sex steroids or SHBG and the dense area substantially, and hence only the crude models are presented (Table 3).

**Table 2:** Mean percentage breast density for quintiles of sex steroids and sex hormone binding globulin (*n *= 775)

	Quintile 1	Quintile 2	Quintile 3	Quintile 4	Quintile 5	*P *value for trend
Oestrone^a^						
Crude	24.7 (22.8–26.7)	22.6 (20.7–24.6)	22.0 (20.3–23.9)	22.3 (20.6–24.0)	20.1 (18.5–21.8)	<0.01
+ BMI	23.6 (21.9–25.5)	21.8 (20.0–23.6)	22.3 (20.6–24.0)	22.6 (21.0–24.2)	21.2 (19.6–22.9)	0.12
Oestradiol^b^						
Crude	24.7 (22.8–26.6)	23.4 (21.6–25.3)	20.8 (19.1–22.6)	22.5 (20.7–24.4)	20.2 (18.5–21.9)	<0.001
+ BMI	23.1 (21.4–24.9)	22.5 (20.8–24.3)	20.7 (19.1–22.4)	23.1 (21.4–25.0)	22.0 (20.3–23.7)	0.58
Free oestradiol^c^						
Crude	24.9 (23.1–26.9)	23.5 (21.7–25.4)	21.6 (19.8–23.4)	22.0 (20.3–23.8)	19.5 (17.9–21.2)	<0.0001
+ BMI	23.1 (21.4–24.9)	22.5 (20.9–24.3)	21.5 (19.8–23.2)	22.6 (20.9–24.4)	21.6 (19.9–23.4)	0.34
Dehydroepiandrosterone sulfate^d^						
Crude	22.7 (20.9–24.6)	21.9 (20.1–23.8)	20.9 (19.1–22.7)	22.8 (21.0–24.6)	23.1 (21.3–25.0)	0.44
+ BMI	22.5 (20.8–24.3)	22.1 (20.4–23.8)	21.2 (19.5–22.9)	22.8 (21.1–24.6)	22.8 (21.1–24.6)	0.54
Androstenedione^e^						
Crude	21.8 (20.0–23.7)	22.9 (21.0–24.7)	22.0 (20.2–23.8)	23.6 (21.8–25.5)	21.1 (19.4–22.9)	0.52
+ BMI	21.1 (19.5–22.9)	22.7 (21.0–24.4)	22.5 (20.8–24.3)	23.5 (21.8–25.3)	21.6 (19.9–23.3)	0.88
Testosterone^f^						
Crude	23.2 (21.4–25.2)	23.8 (22.0–25.7)	22.2 (20.4–24.1)	21.7 (20.0–23.5)	20.5 (18.8–22.2)	0.01
+ BMI	21.9 (20.2–23.7)	23.4 (21.7–25.2)	22.2 (20.5–24.0)	22.1 (20.4–23.8)	21.7 (20.1–23.5)	0.48
Free testosterone^g^						
Crude	26.0 (24.1–28.0)	22.9 (21.1–24.8)	21.1 (19.4–22.9)	21.7 (20.0–23.5)	19.8 (18.1–21.5)	<0.0001
+ BMI	24.2 (22.5–26.1)	22.1 (20.4–23.8)	21.0 (19.4–22.7)	22.5 (20.8–24.2)	21.6 (19.9–23.3)	0.13
Sex hormone binding globulin^h^						
Crude	19.3 (17.6–21.0)	20.1 (18.5–21.9)	22.8 (21.0–24.6)	23.0 (21.2–24.9)	26.2 (24.4–28.2)	<0.0001
+ BMI	21.4 (19.7–23.2)	20.8 (19.2–22.5)	22.4 (20.7–24.1)	22.4 (20.7–24.1)	24.4 (22.6–26.3)	<0.01
+ BMI and WHR	21.9 (20.2–23.8)	21.4 (19.7–23.1)	22.2 (20.6–23.9)	22.2 (20.5–23.9)	23.7 (21.9–25.5)	0.11

Data presented as the mean (95% confidence interval). BMI, body mass index; WHR, waist–hip ratio. ^a^Quintile cutoff points: 7.45, 12, 15, 20 and 29 pg/ml. ^b^Quintile cutoff points: 4.45, 6.4, 8.2, 10.0 and 14 pg/ml. ^c^Quintile cutoff points: 0.12, 0.18, 0.24, 0.31 and 0.45 pg/ml. ^d^Quintile cutoff points: 188, 333, 479, 666 and 1,041 ng/ml. ^e^Quintile cutoff points: 0.19, 0.36, 0.50, 0.67 and 1.10 ng/m. ^f^Quintile cutoff points: 0.13, 0.20, 0.26, 0.33 and 0.47 ng/m. ^g^Quintile cutoff points: 2.29, 3.98, 5.48, 7.50 and 11.36 pg/ml. ^h^Quintile cutoff points: 6.2, 15, 21, 28 and 43 nmol/l.

**Table 3:** Mean dense breast area for quintiles of sex steroids and sex hormone binding globulin (*n *= 775)

	Quintile 1	Quintile 2	Quintile 3	Quintile 4	Quintile 5	*P *value for trend
Oestrone^a^	27.4 (25.3–29.5)	27.3 (25.1–29.6)	27.0 (25.0–29.1)	26.5 (24.6–28.5)	25.2 (23.3–27.2)	0.09
Oestradiol^b^	27.9 (25.8–30.0)	27.7 (25.7–29.8)	24.3 (22.4–26.3)	27.2 (25.2–29.4)	26.1 (24.2–28.1)	0.27
Free oestradiol^c^	27.8 (25.7–29.9)	27.3 (25.2–29.4)	26.0 (24.0–28.0)	26.3 (24.3–28.3)	25.9 (23.9–28.0)	0.20
Dehydroepiandrosterone sulfate^d^	27.8 (25.7–29.9)	26.0 (24.0–28.1)	26.5 (24.5–28.6)	26.2 (24.2–28.3)	26.7 (24.7–28.8)	0.68
Androstenedione^e^	26.8 (24.8–28.9)	27.1 (25.1–29.3)	25.5 (23.5–27.6)	28.2 (26.2–30.3)	25.5 (23.5–27.5)	0.44
Testosterone^f^	27.1 (25.0–29.2)	27.5 (25.5–29.6)	25.9 (23.9–28.0)	26.3 (24.3–28.3)	26.4 (24.4–28.5)	0.53
Free testosterone^g^	28.7 (26.6–30.8)	25.6 (23.7–27.7)	25.3 (23.4–27.4)	27.5 (25.4–29.6)	26.1 (24.1–28.1)	0.36
Sex hormone binding globulin^h^	26.3 (24.2–28.4)	26.2 (24.2–28.2)	25.5 (23.6–27.5)	27.7 (25.6–29.8)	27.5 (25.5–29.7)	0.22

Data presented as the mean (95% confidence interval). ^a^Quintile cutoff points: 7.45, 12, 15, 20 and 29 pg/ml. ^b^Quintile cutoff points: 4.45, 6.4, 8.2, 10.0 and 14 pg/ml. ^c^Quintile cutoff points: 0.12, 0.18, 0.24, 0.31 and 0.45 pg/ml. ^d^Quintile cutoff points: 188, 333, 479, 666 and 1,041 ng/ml. ^e^Quintile cutoff points: 0.19, 0.36, 0.50, 0.67 and 1.10 ng/m. ^f^Quintile cutoff points: 0.13, 0.20, 0.26, 0.33 and 0.47 ng/m. ^g^Quintile cutoff points: 2.29, 3.98, 5.48, 7.50 and 11.36 pg/ml. ^h^Quintile cutoff points: 6.2, 15, 21, 28 and 43 nmol/l.

**Table 4:** Mean nondense breast area for quintiles of sex steroids and sex hormone binding globulin (*n *= 775)

	Quintile 1	Quintile 2	Quintile 3	Quintile 4	Quintile 5	*P *value for trend
Oestrone^a^						
Crude	85.5 (79.7–91.4)	93.8 (87.5–100.3)	97.5 (91.5–103.6)	95.2 (89.6–101.0)	101.4 (95.5–107.6)	<0.001
+ BMI	90.9 (86.1–95.9)	98.6 (93.4–104.0)	96.1 (91.3–101.0)	93.7 (89.1–98.3)	94.9 (90.1–99.7)	0.74
Oestradiol^b^						
Crude	87.0 (81.3–92.8)	91.6 (85.8–97.6)	94.7 (88.7–100.8)	95.6 (89.6–101.8)	104.9 (98.9–111.2)	<0.0001
+ BMI	95.5 (90.6–100.6)	97.0 (92.1–101.9)	95.5 (90.6–100.4)	91.9 (87.1–96.8)	93.8 (89.0–98.7)	0.36
Free oestradiol^c^						
Crude	85.1 (79.6–90.8)	90.3 (84.6–96.2)	96.2 (90.3–102.2)	94.4 (88.6–100.4)	108.7 (102.4–115.1)	<0.0001
+ BMI	95.1 (90.2–100.2)	95.6 (90.7–100.5)	96.8 (92.0–101.8)	90.8 (86.1–95.7)	95.3 (90.3–100.4)	0.69
Dehydroepiandrosterone sulfate^d^						
Crude	96.0 (90.1–102.2)	95.6 (89.7–101.8)	101.0 (94.9–107.3)	91.3 (85.5–97.4)	90.1 (84.3–96.0)	0.06
+ BMI	97.2 (92.3–102.1)	94.7 (89.9–99.6)	99.3 (94.4–104.3)	91.1 (86.4–95.9)	91.6 (86.9–96.4)	0.05
Androstenedione^e^						
Crude	97.9 (91.8–104.2)	93.8 (87.9–99.9)	92.8 (86.8–99.0)	92.7 (86.9–98.6)	96.8 (90.8–103.0)	0.99
+ BMI	101.9 (96.9–107.0)	95.0 (90.2–99.9)	89.8 (85.1–94.7)	93.2 (88.6–97.9)	94.0 (89.3–98.9)	0.10
Testosterone^f^						
Crude	90.8 (84.8–96.9)	90.7 (85.0–96.6)	93.4 (87.5–99.6)	95.8 (89.9–101.8)	103.3 (97.1–109.6)	<0.01
+ BMI	98.1 (93.0–103.3)	92.8 (88.1–97.6)	93.5 (88.7–98.4)	93.9 (89.2–98.7)	95.7 (90.8–100.6)	0.82
Free testosterone^g^						
Crude	83.6 (78.2–89.3)	88.4 (82.8–94.3)	96.2 (90.3–102.2)	100.3 (94.3–106.5)	106.2 (100.0–112.6)	<0.0001
+ BMI	92.9 (88.1–97.9)	93.2 (88.4–98.1)	96.8 (92.0–101.8)	95.7 (90.9–100.7)	95.0 (90.0–100.0)	0.53
Sex hormone binding globulin^h^						
Crude	110.7 (104.4–117.2)	105.5 (99.5–111.7)	88.6 (83.1–94.2)	92.7 (87.1–98.5)	79.2 (74.0–84.5)	<0.0001
+ BMI	97.0 (91.9–102.3)	101.0 (96.0–106.0)	90.9 (86.3–95.6)	96.7 (91.9–101.6)	88.6 (84.0–93.4)	<0.01
+ BMI and WHR	94.3 (89.2–99.4)	98.8 (94.0–103.8)	91.6 (87.1–96.2)	97.4 (92.7–102.3)	91.3 (86.6–96.1)	0.32

Data presented as the mean (95% confidence interval). BMI, body mass index; WHR, waist–hip ratio. ^a^Quintile cutoff points: 7.45, 12, 15, 20 and 29 pg/ml. ^b^Quintile cutoff points: 4.45, 6.4, 8.2, 10.0 and 14 pg/ml. ^c^Quintile cutoff points: 0.12, 0.18, 0.24, 0.31 and 0.45 pg/ml. ^d^Quintile cutoff points: 188, 333, 479, 666 and 1,041 ng/ml. ^e^Quintile cutoff points: 0.19, 0.36, 0.50, 0.67 and 1.10 ng/m. ^f^Quintile cutoff points: 0.13, 0.20, 0.26, 0.33 and 0.47 ng/m. ^g^Quintile cutoff points: 2.29, 3.98, 5.48, 7.50 and 11.36 pg/ml. ^h^Quintile cutoff points: 6.2, 15, 21, 28 and 43 nmol/l.

The inverse associations of circulating levels of both oestrogens and testosterone with the percentage density were no longer apparent when adjusted for BMI (Table 2). DHEAS and androstenedione were not related to percentage density. Women with high SHBG levels showed significantly higher percentage density than women with low SHBG levels (Table 2). This association was still significant after correction for BMI, but was no longer significant after further adjustment for WHR. High oestrone levels were associated with a lower dense area, but the relation was not statistically significant. None of the other sex steroids or SHBG was related to the dense area (Table 3). Before adjustment, higher levels of both oestrogens and testosterone were significantly associated with higher nondense area. After adjusting for BMI, however, these associations disappeared. (Table 4). High levels of DHEAS were borderline significantly associated with lower nondense area, irrespective of adjustment for BMI (Table 4). High levels of SHBG were very strongly associated with low nondense area (Table 4). This relationship became less strong after corrections for BMI and disappeared after additional adjustment for WHR.

When we stratified the analyses according to HT ever use (yes/no), results were highly comparable between the groups.

## Discussion

The significant inverse associations between both oestrogens and testosterone with percentage beast density disappeared after adjustment for BMI. No relationships were found between sex steroids and the absolute measure of mammographic density, the dense area. High levels of SHBG were associated with high percentage density and a low nondense area. These relations disappeared after corrections for BMI and for WHR.

An advantage of the present study is its size – with 775 participants, our study is larger than all previous studies [14-17] except that of Warren and colleagues [18]. Another advantage is the fact that the blood sample we used for hormone measurements was drawn at the same day as mammography was performed for most of the women (98.3%). In addition to the measurement of total hormone levels, we also calculated free levels of oestradiol and testosterone, because it is the unbound hormone fraction that is expected to affect the target (dense) tissue most clearly. The free levels used in the present study were not directly measured but were calculated from total plasma concentrations of oestradiol, testosterone and SHBG, using theoretical calculations [25]. In a validation study, Rinaldi and colleagues. showed high correlation between measured concentrations and calculated concentrations of both free oestradiol and free testosterone in postmenopausal women (*r *= 0.84 and *r *= 0.76, respectively) [30]. If misclassification due to measurement errors of the sex steroids or the breast measures has occurred, this most probably has been nondifferential. This misclassification may have led to bias to the null relation, but we do not believe this is an explanation for the null results we found with the dense area.

To our best knowledge, seven previous studies on sex steroids and breast density have been published [14-18,31,32], five of which reported results on postmenopausal women [14-18]. With the exception of the study by Boyd and colleagues [15], all these studies reported results with the relative measure of breast density (percentage density) only – although Aiello and colleagues and Tamimi and colleagues mentioned that analyses with absolute breast density gave similar results [16,17]. Although Boyd and colleagues found slightly stronger effects with percentage breast density than with the dense area [15], the dense area may be a better marker of breast cancer risk as it is the absolute amount of glandular and stromal (dense) tissue, which is regarded as the target tissue for breast cancer [27,28]. None of the previous studies on sex steroids and breast density presented results for the nondense area, which is part of the denominator to calculate the percentage breast density.

In the present study, crude models show both oestrone and oestradiol to be inversely related to the percentage density. After correction for BMI these relations were no longer apparent. Associations with free oestradiol were in the same direction and were somewhat stronger. Three other studies also found the crude inverse relation between oestradiol and percentage density, but only in the study by Boyd and colleagues did this association remain statistically significant after adjustments for confounders, although only with free oestradiol [15,18]. This inverse relationship appeared to be stronger for the percentage density than for the absolute dense area. Aiello and colleagues only presented results of multivariate analyses, which showed oestrogens not to be related to percentage density [16]. Only Greendale and colleagues found the opposite result, with a positive association between oestrogen levels and percentage density, which even became stronger and statistically significant after correcting for confounding variables [14]. We do not know what caused this different finding by Greendale and colleagues. A relatively large percentage of their study population consisted of former HT users and the average time since last use of these compounds was smaller compared with other studies. It is not known what time period is needed for a complete washout of the effect of exogenous hormones on both endogenous sex steroid levels and breast density. Results of ever users should therefore be interpreted cautiously. In the present study, only 14% of the study participants were former HT users and the median time since last HT use was 5 years (interquartile range, 2–10 years). When we stratified the analyses to HT ever use, oestrogen levels were significantly inversely related to both percentage breast density and dense area, which was not seen in the subgroup of never HT users. The difference in effect between never HT users and ever HT users was not seen with any of the other sex steroids or SHBG, and the results with oestrone levels may be chance.

Our result of a positive association between circulating SHBG levels and the percentage breast density are in line with four previous studies [14,15,17,18]. As in the present study, the crude effect in most of these studies was very explicit, but became weaker after correction for confounding factors, especially BMI or other measures of total body fat. In our study, women with high SHBG levels had slightly, but nonsignificantly higher dense area and smaller nondense area. The apparently positive association between SHBG levels and percentage breast density or dense area seems in contradiction with the inverse relation between these SHBG levels and breast cancer risk that has been described in many epidemiologic studies [12,13]. A possible explanation was offered by Greendale and colleagues, who suggested a difference in SHBG-receptor-mediated effects between normal and cancerous breast tissue [14].

Studying so many associations raises the question of whether multiple testing has resulted in false positive results. The positive crude relations between both oestrogens and testosterone and the nondense area, however, were expected. After menopause, circulating oestrogen levels reflect the conversion of androgens to oestrogens in adipose tissue. Most of the circulating testosterone is also converted in the adipose tissue from androstenedione [33]. Oestrogens and testosterone are therefore strongly correlated with BMI, as is the nondense (fat) area. These relationships also explain the crude inverse associations between both oestrogens and testosterone with percentage density, as the nondense area is in the denominator to calculate this relative measure of mammographic density. After correction for BMI the relations between oestrogens and testosterone and both the percentage density and the nondense area disappeared.

Circulating progesterone levels were not available in the present study. As combined oestrogen and progesterone HT use exerts stronger effects on breast density than oestrogen alone treatment [34-37], progesterone levels may be associated with breast density. Although only one [15] of the four studies [14,15,17,18] researching this association found a significant increase in breast density, progesterone levels and combined oestrogen and progesterone levels may affect breast density.

## Conclusion

After studying our results and the results of five previous studies on sex steroids and postmenopausal breast density, we conclude that there is no major proof for such associations in postmenopausal women, at least not to the same extent as the associations between sex steroid levels and breast cancer risk. Some of the previous studies were relatively small, but the study by Warren and colleagues [18] as well as our own study had enough power to detect such effects. The relationship between sex steroids and breast cancer risk as described in the literature does not seem to be explained by a change in mammographic density.

## Abbreviations

BMI = body mass index; DHEAS = dehydroepiandrosterone sulfate; EPIC = European Prospective Investigation into Cancer and Nutrition; HT = hormone therapy; SHBG = sex hormone binding globulin; WHR = waist–hip ratio.

## Competing interests

The authors declare that they have no competing interests.

## Authors' contributions

MV participated in the design of the study, performed the statistical analyses and drafted the manuscript. PHMP conceived of the study, participated in the design of the study and helped to draft the manuscript. PAHvN participated in the design of the study and helped to draft the manuscript. YTvdS conceived of the study, participated in the design and coordination of the study and helped to draft the manuscript. DEG conceived of the study, participated in the design of the study and helped to draft the manuscript. CHvG conceived of the study, participated in the design and coordination of the study, helped to perform the statistical analyses and helped to draft the manuscript. All authors read and approved the final manuscript.

## References

[B1] Byng JW, Boyd NF, Fishell E, Jong RA, Yaffe MJ (1994). The quantitative analysis of mammographic densities. Phys Med Biol.

[B2] Boyd NF, Rommens JM, Vogt K, Lee V, Hopper JL, Yaffe MJ, Paterson AD (2005). Mammographic breast density as an intermediate phenotype for breast cancer. Lancet Oncol.

[B3] McCormack VA, dos Santos Silva I (2006). [AU Query: Provide full last name and initials of all authors]: Breast density and parenchymal patterns as markers of breast cancer risk: a meta-analysis. Cancer Epidemiol Biomarkers Prev.

[B4] McTiernan A, Martin CF, Peck JD, Aragaki AK, Chlebowski RT, Pisano ED, Wang CY, Brunner RL, Johnson KC, Manson JE (2005). Estrogen-plus-progestin use and mammographic density in postmenopausal women: women's health initiative randomized trial. J Natl Cancer Inst.

[B5] Greendale GA, Reboussin BA, Slone S, Wasilauskas C, Pike MC, Ursin G (2003). Postmenopausal hormone therapy and change in mammographic density. J Natl Cancer Inst.

[B6] Chlebowski RT, Hendrix SL, Langer RD, Stefanick ML, Gass M, Lane D, Rodabough RJ, Gilligan MA, Cyr MG, Thomson CA (2003). Influence of estrogen plus progestin on breast cancer and mammography in healthy postmenopausal women: the Women's Health Initiative Randomized Trial. JAMA.

[B7] Boyd NF, Martin LJ, Li Q, Sun L, Chiarelli AM, Hislop G, Yaffe MJ, Minkin S (2006). Mammographic density as a surrogate marker for the effects of hormone therapy on risk of breast cancer. Cancer Epidemiol Biomarkers Prev.

[B8] Brisson J, Brisson B, Cote G, Maunsell E, Berube S, Robert J (2000). Tamoxifen and mammographic breast densities. Cancer Epidemiol Biomarkers Prev.

[B9] Cuzick J, Warwick J, Pinney E, Warren RM, Duffy SW (2004). Tamoxifen and breast density in women at increased risk of breast cancer. J Natl Cancer Inst.

[B10] Shah NR, Borenstein J, Dubois RW (2005). Postmenopausal hormone therapy and breast cancer: a systematic review and meta-analysis. Menopause.

[B11] Clavel-Chapelon F, Gerber M (2002). Reproductive factors and breast cancer risk. Do they differ according to age at diagnosis?. Breast Cancer Res Treat.

[B12] Key T, Appleby P, Barnes I, Reeves G (2002). Endogenous sex hormones and breast cancer in postmenopausal women: reanalysis of nine prospective studies. J Natl Cancer Inst.

[B13] Kaaks R, Rinaldi S, Key TJ, Berrino F, Peeters PH, Biessy C, Dossus L, Lukanova A, Bingham S, Khaw KT (2005). Postmenopausal serum androgens, oestrogens and breast cancer risk: the European prospective investigation into cancer and nutrition. Endocr Relat Cancer.

[B14] Greendale GA, Palla SL, Ursin G, Laughlin GA, Crandall C, Pike MC, Reboussin BA (2005). The association of endogenous sex steroids and sex steroid binding proteins with mammographic density: results from the Postmenopausal Estrogen/Progestin Interventions Mammographic Density Study. Am J Epidemiol.

[B15] Boyd NF, Stone J, Martin LJ, Jong R, Fishell E, Yaffe M, Hammond G, Minkin S (2002). The association of breast mitogens with mammographic densities. Br J Cancer.

[B16] Aiello EJ, Tworoger SS, Yasui Y, Stanczyk FZ, Potter J, Ulrich CM, Irwin M, McTiernan A (2005). Associations among circulating sex hormones, insulin-like growth factor, lipids, and mammographic density in postmenopausal women. Cancer Epidemiol Biomarkers Prev.

[B17] Tamimi RM, Hankinson SE, Colditz GA, Byrne C (2005). Endogenous sex hormone levels and mammographic density among postmenopausal women. Cancer Epidemiol Biomarkers Prev.

[B18] Warren R, Skinner J, Sala E, Denton E, Dowsett M, Folkerd E, Healey CS, Dunning A, Doody D, Ponder B (2006). Associations among mammographic density, circulating sex hormones, and polymorphisms in sex hormone metabolism genes in postmenopausal women. Cancer Epidemiol Biomarkers Prev.

[B19] Riboli E, Kaaks R (1997). The EPIC Project: rationale and study design. European Prospective Investigation into Cancer and Nutrition. Int J Epidemiol.

[B20] Riboli E, Hunt KJ, Slimani N, Ferrari P, Norat T, Fahey M, Charrondiere UR, Hemon B, Casagrande C, Vignat J (2002). European Prospective Investigation into Cancer and Nutrition (EPIC): study populations and data collection. Public Health Nutr.

[B21] Boker LK, Van Noord PA, van der Schouw YT, Koot NV, Bueno De Mesquita HB, Riboli E, Grobbee DE, Peeters PH (2001). Prospect-EPIC Utrecht: study design and characteristics of the cohort population. European Prospective Investigation into Cancer and Nutrition. Eur J Epidemiol.

[B22] Ocke MC, Bueno-de-Mesquita HB, Pols MA, Smit HA, van Staveren WA, Kromhout D (1997). The Dutch EPIC food frequency questionnaire. II. Relative validity and reproducibility for nutrients. Int J Epidemiol.

[B23] Ocke MC, Bueno-de-Mesquita HB, Goddijn HE, Jansen A, Pols MA, van Staveren WA, Kromhout D (1997). The Dutch EPIC food frequency questionnaire. I. Description of the questionnaire, and relative validity and reproducibility for food groups. Int J Epidemiol.

[B24] Onland-Moret NC, Peeters PH, van der Schouw YT, Grobbee DE, Van Gils CH (2005). Alcohol and endogenous sex steroid levels in postmenopausal women: a cross-sectional study. J Clin Endocrinol Metab.

[B25] Vermeulen A, Verdonck L, Kaufman JM (1999). A critical evaluation of simple methods for the estimation of free testosterone in serum. J Clin Endocrinol Metab.

[B26] Byng JW, Boyd NF, Little L, Lockwood G, Fishell E, Jong RA, Yaffe MJ (1996). Symmetry of projection in the quantitative analysis of mammographic images. Eur J Cancer Prev.

[B27] Trichopoulos D, Lipman RD (1992). Mammary gland mass and breast cancer risk. Epidemiology.

[B28] Albanes D, Winick M (1988). Are cell number and cell proliferation risk factors for cancer?. J Natl Cancer Inst.

[B29] Haars G, Van Noord PA, Van Gils CH, Grobbee DE, Peeters PH (2005). Measurements of breast density: no ratio for a ratio. Cancer Epidemiol Biomarkers Prev.

[B30] Rinaldi S, Geay A, Dechaud H, Biessy C, Zeleniuch-Jacquotte A, Akhmedkhanov A, Shore RE, Riboli E, Toniolo P, Kaaks R (2002). Validity of free testosterone and free estradiol determinations in serum samples from postmenopausal women by theoretical calculations. Cancer Epidemiol Biomarkers Prev.

[B31] Noh JJ, Maskarinec G, Pagano I, Cheung LW, Stanczyk FZ (2006). Mammographic densities and circulating hormones: a cross-sectional study in premenopausal women. Breast.

[B32] Meyer F, Brisson J, Morrison AS, Brown JB (1986). Endogenous sex hormones, prolactin, and mammographic features of breast tissue in premenopausal women. J Natl Cancer Inst.

[B33] Simpson ER (2003). Sources of estrogen and their importance. J Steroid Biochem Mol Biol.

[B34] Collaborative Group on Hormonal Factors in Breast Cancer (1997). Breast cancer and hormone replacement therapy: collaborative reanalysis of data from 51 epidemiological studies of 52,705 women with breast cancer and 108,411 women without breast cancer. Collaborative Group on Hormonal Factors in Breast Cancer. Lancet.

[B35] Rossouw JE, Anderson GL, Prentice RL, LaCroix AZ, Kooperberg C, Stefanick ML, Jackson RD, Beresford SA, Howard BV, Johnson KC (2002). Risks and benefits of estrogen plus progestin in healthy postmenopausal women: principal results from the Women's Health Initiative randomized controlled trial. JAMA.

[B36] Chlebowski RT, Hendrix SL, Langer RD, Stefanick ML, Gass M, Lane D, Rodabough RJ, Gilligan MA, Cyr MG, Thomson CA (2003). Influence of estrogen plus progestin on breast cancer and mammography in healthy postmenopausal women: the Women's Health Initiative Randomized Trial. JAMA.

[B37] van Duijnhoven FJ, Peeters PH, Warren RM, Bingham SA, Van Noord PA, Monninkhof EM, Grobbee DE, Van Gils CH (2007). Postmenopausal hormone therapy and changes in mammographic density. J Clin Oncol.

